# Covid-19 apps, Corona vaccination apps and data “ownership”

**DOI:** 10.1007/s12689-022-00097-7

**Published:** 2022-11-23

**Authors:** Sjef van Erp

**Affiliations:** 1 Emeritus Professor of Civil Law and European Private Law Maastricht University, Maastricht, The Netherlands; 2 Visiting Professor, Trento University, Trento, Italy; 3 President International Association of Legal Science, Paris, France; 4 Deputy-Justice Court of Appeals ‘s-Hertogenbosch, ‘s Hertogenbosch, The Netherlands

**Keywords:** Health data, Data ownership, Covid-19 apps, Vaccination apps, Data management

## Abstract

Already before the present Covid-19 health crisis an emerging trend could be seen towards offering health services from a distance, called “e-health”. This trend, like so many other developments towards digitalisation of our societies, received a considerable impetus because of the Covid-19 crisis. First, the rise of Covid-19 tracing (and/or tracking) apps and now to be followed by the advance of Corona vaccination apps has made us aware of the benefits which e-health may bring, particularly in a situation where distance means safety. The apps contain very personal information and, consequently, have provoked questions as to whether the apps sufficiently protect a person’s right to privacy and data protection as safeguarded by the EU’s General Data Protection Regulation. The nature of the data, however, is such that also questions as to the importance of access by public health authorities in the public interest can be asked. Also, although commercial, but still important for developing and producing vaccines, for the pharmaceutical industry the data are important. The result is a conflict particularly between entitlement to privacy protection and the general interest, causing questions to be asked about which interest has priority. It might very well be, however, that this question, asked as such, is beside the point. Given that data are non-rivalrous and non-depletable, because they can be copied and copied, questions about which entitlement has priority cannot be answered in absolute terms. Rights regarding data depend upon who at a particular time has control over the data, who else has control and what control between all those involved then means. Looking at who has which right to data one can see an entitlement paradigm surfacing which is multi-perspective, relative and dynamic. Calling data entitlement “ownership” is not a reference to ownership in the traditional sense of the word, but to management. To decide what management in a particular situation means interest balancing exercises must be made. These exercises will change over time, as accordingly will the answer to the question who is “owner” of data in Covid-19 and Corona vaccination apps.

## Introduction: e-health

The Covid-19 pandemic has shown us, once more, how interconnected we are across continents. We already were aware of how global the economy had become, but because of the pandemic we now all realise far too well that this interconnectedness not only may bring us substantial benefits but also substantial risks. Although because of the pandemic we now, at least temporarily, travel less and physically remain more at a distance than we were used to, at a more technical level our interconnectedness increased. Where online meetings and online classrooms were still an exception a year or so ago, they are now widely used and may have a lasting impact on how we get into contact with one another. But also how we react to the pandemic shows this almost contradictory approach: We keep one another at a safe distance, but at the same time we replace physical contact with virtual interaction. An example are online medical consultations with, for example, your family doctor. Still, any diagnosis requires the gathering of data, comprising information about a particular person. Where it used to be the case that these data needed to be collected through physical examination only, we now have different tools at our disposition. This has resulted in what is being called “e-health”: the use of, for example, applications (“apps”) on a person’s telephone to assist a nurse or doctor in monitoring and treating a patient.^1^ In this way someone can be observed from a distance and does not have to go to a hospital weekly to have, for example, his or her blood pressure checked, because the patient after checking the blood pressure him/herself adds these data to a health app, which then transmits the data to the hospital. Until the Covid-19 pandemic these developments were frequently more of an experimental nature than anything else, but the pandemic made us realise that e-health enables distance diagnosing, which protects everyone involved. Being monitored by an e-health app on your mobile phone allows treating patients in such a way that patients can stay at home while doctors continue working in their practice, without having to travel and getting into close physical contract, but still giving patients the care they need. Several e-health tools are now available, but the focus of this contribution is on an e-health tool that was developed particularly in reaction to the Covid-19 pandemic: the Covid-19 tracing and/or tracking apps and an e-health tool that is now being developed, tested and already used in some countries that provides information on whether you have been vaccinated, tested negative or have Covid-19 antibodies: the Covid vaccine or immunity passport.^2^ Both apps, as in fact all e-health apps, provide data about someone’s physical integrity and accordingly contain very personal information. At the same time it is precisely this very information which might be vitally important for someone else’s health or, for that matter, the health of a whole population. Very fundamental questions can be asked as to who is entitled to (“owns”) that information and whether some type of priority can be distinguished between “owners” of data or whether perhaps an overriding public interest should be recognised.^3^ Before offering an answer to those questions, first the legal status of data will be discussed and whether any rights regarding data (“data rights”), given that data are non-rivalrous and non-depletable, are of a different nature than traditional private law rights.

## Data: A mirror or creator of reality?

Within only a few decades we went from information in oral and written form to information in the form of data. The World Economic Forum calls this process the Third and Fourth Industrial Revolution. When mentioning the Industrial Revolution generally reference is made to a period of mass production and mass consumption at the end of the 18th, beginning nineteenth century, made possible by the invention of the steam engine and the following mechanisation of production. This development got accelerated by the invention of electricity and the innovations made possible by this new technique, thus creating the Second Industrial Revolution. From the middle of the twentieth century digitalisation and the building of databases began to develop with the help of Information Technology (IT) and electronics (the Third Industrial Revolution). Since the end of the last century until today this is followed by a rapid growth of the volume of data (“big data”) and the interconnectivity and interoperability of data (the “Internet” and the exponentially growing number and use of mobile devices, such as cell phones), the development of data analysis as a science (“data science”), an increasing use of new types of software making it possible to replace money or other assets with digital tools (Distributed Ledger Technology (DLT), such as blockchain, creating “crypto-currencies” and “tokens”, turning into an “Interchain”: the Internet of blockchains), self-executing computer programmes (or “smart” contracts), collecting data with sensors (“sensorisation” and the “Internet of Things”) and the progress of software that is able to develop itself in its use by advanced analysis of data input (“Artificial Intelligence”). These last developments are called the Fourth Industrial Revolution. From a legal perspective (but not only from that perspective) all of this is creating a what might well be perceived as a “perfect storm”: several developments coming together at the same moment, creating a new reality until now unknown to the law.

This new digital economy is raising several very fundamental legal questions, one of them being who is entitled against whom regarding data and another being what does “entitlement” then mean? As such this is an overarching question which provokes a debate about privacy (a data subject having certain public law rights against a data controller about that subject’s personal data), the nature of data sales contracts (what does selling data legally mean?) and how to attribute as well as distribute these entitlements and any possibly resulting economic benefits. Traditionally, regarding entitlements the Civil Law makes two very basic distinctions. First, between public and private law, and second within private law between personal and real rights. In two very interesting publications Lian Yuming introduced the possibility of having a category of rights which are neither public, nor private and personal, nor real, but encompass all of these aspects. The name given to these rights would be “data rights”.^4^ As happens so often with new visions on the future, that same approach can be found in a joint project of the American Law Institute (ALI) with its European counterpart the European Law Institute (ELI) aimed at formulating Principles for a Data Economy.^5^ Also in the approach taken in this project the data economy is to be governed by data rights, defined as: “legally protected interests that arise from the very nature of data as information recorded in any form or medium.”^6^ In this contribution I will not attempt to evaluate this approach towards the development of a new category of subjective rights, although I can only express that a radically new methodology, founded on existing law and the drawing of analogies while learning from acquired wisdom, is most probably the only way to deal in a holistically with the legal aspects of data. My focus, however, will be more on entitlements which we traditionally call property rights: A right which a subject has against a considerable and relevant group of other subjects regarding an object.

From a property law perspective the data economy gives rise to a great variety of legal problems. Because data are non-rivalrous and cannot be depleted they do not fit very well (perhaps, depending upon the particular legal system: not at all) in the existing categories of legal objects. From a general linguistic view, dictionaries have different ways of describing what an “object” is. Generally speaking, an object is “something mental or physical toward which thought, feeling, or action is directed”.^7^ The law, more particularly property law, only accepts certain objects as legally relevant. Mostly, because these objects have economic value. Generally speaking, this can only be seen indirectly, or perhaps better formulated, in an implicit manner. One of the leading principles of property law is the numerus clausus principle. According to this tenet of property law the number and content of property rights are restricted (substantive aspect), the procedure how to create, transfer and terminate a property right is of a mandatory nature (procedural aspect), those who can be subjects are limited (in general: natural and legal persons; the subjective aspect) and the objects of such rights are limited by including a description of the object in the definition of the right (objective aspect).^8^ These four aspects are interrelated. Specific subjects might only be entitled to specific objects, depending upon who the subject is or what the object is. An non-incorporated association may, for example, not be entitled to own immovable property. What a particular property right implies might also depend upon the object. Ownership of immovable property could result in the application of neighbour law, this is not to be expected regarding ownership of a car. Ownership of physical property will differ from ownership of (in some legal systems: entitlement to) monetary claims. It can overall be stated that legal systems accept physical things (Immovables and movables: land, houses, cars etc.) as a legal object of property rights. Legal systems also accept intangibles as legal objects (for example monetary claims). Products resulting from human creativity were seen as problematic, but that was solved by creating a separate legal area focussing on intellectual property. As we already saw, it is argued that perhaps we should do the same with data: create a separate legal area focussing on data as an object, data subjects, data rights and specific mandatory procedures regarding such rights.

The difficulty with creating such a separate legal area, which we might call “data rights law”, is that the nature of data, as being non-rivalrous and non-depletable, is such that any data rights cannot be classified as belonging to any of the traditional categories of rights. Limiting myself to a property perspective data rights, as proposed, will be quasi-proprietary as giving their holder a right to an exclusive use against some, but not against all. Also the right will evolve dynamically over time, depending on who at a particular moment will have a personal (privacy), commercial (economic) or general (public) interest. This is why I defend the view that the debate about data ownership is not about ownership in the traditional sense of the word, but about management of data and management rights given to different types of managers with different rights of control: access, exclude, use, modify, delete, port and transfer.

However, in our quest for a data governance structure we seem to forget that by focussing on, what might perhaps be called, the design of rights, like we traditionally do when formulating a property right as part of the numerus clausus, we also define the object of that right. By doing so we create a legal object. But not only that. Next to defining a legal object we also express which subject will have a data right against which other subjects and we attribute the right. In other words: the creation of data rights not only mirrors a reality of de facto wealth distribution, but it also de iure creates such a distribution. This is what makes the debate so complicated, as it is not only strictly legal, but also economic and political. It is legal as we try to formulate a normative framework that functions fair and equitable with regard to these new objects: data. But it is also economic, because by doing so at this initial stage of data law development we also distribute wealth, and it is that aspect that also makes this a political question, furthermore given that in the general interest also the government might be entitled to a data right. This entitlement of governments to data could be very well compared to the more traditional concept of dominium eminens, giving a state the right to expropriate or seize a person’s assets. Such data sovereignty of states (differing from data sovereignty in the sense of entitlement by an individual to protection of personal data) is more and more claimed. A striking example is Australia which in Sect. 94 ZC its Privacy Amendment (Public Health Contact Information) Act 2020 enacted that “COVID app data is the property of the Commonwealth, and remains the property of the Commonwealth even after it is disclosed to, or used by: (a) a State or Territory health authority; or (b) any other person or body (other than the Commonwealth or an authority of the Commonwealth).”^9^ With such a provision expropriation is even no longer needed, because the state already is the owner of these data. Also the European Union is moving towards the claim that certain data belong to the European Union (or at least, so I would like to add, its Member-States), as the European Commission laid down in its Digital Compass 2030.^10^ It seems as if the European ambition is going further than what Australia enacted, as it targets not only the data themselves, but also the whole data infrastructure. The European Commission puts “special emphasis on a European Cloud, leadership in ethical artificial intelligence, a secure digital identity for all, and vastly improved data, supercomputer and connectivity infrastructures”.^11^ We can see that gradually, given the enormous impact which data have on our way of life, governments cannot but accept that some control over these data is unavoidable and even necessary to realise their policies. These could be aimed at privacy protection, protecting public health or promoting the sharing of data, for example among businesses or between governments and businesses.^12^

In the next part of this contribution I will focus on two specific examples to show the difficulties with which legal systems, especially their traditional rules on property law, are faced when confronted with our new hybrid (real and virtual) world. These examples concern data rights questions raised by the use of Corona or Corona vaccination apps. An advantage of this approach is that the problems and the resulting questions become more concrete and make us realise more directly how the rather abstract idea of data rights might function in specific cases and situations.

## Covid-19 apps: tracing and tracking

Given the developments towards e-health it can hardly come as a surprise that immediately when the impact of the pandemic became clear digital tools were being developed to counter the spread of the disease. Transmission in the case of Covid-19 occurs by human contact (for example shaking hands or through breathing aerosols). So, immediately the idea came up to see if, with the help of mobile devices such as someone’s mobile phone, relevant contacts of an infected person could be detected. Such information could then be used to inform them. In this way mobile contact tracing could become an e-health tool in combatting the pandemic. Mobile phone providers already follow their users by tracking them, which can be done by using various technical means. A user is followed real time, because a provider needs to know where the phone physically is in order for making it technically possible to connect it with the nearest base station (mobile transmitter tower). Tracking can also happen with the location and GPS facilities on mobile phones. However, if the Corona app would track mobile phone users this would be in violation of the EU’s General Data Protection Regulation (GDPR), as the contacts data clearly would be seen as personal in the sense of article 4(1) GDPR and, consequently, the mobile phone user would qualify as a “data subject” in the sense of the GDPR and would be entitled to data protection.^13^ This is why a choice was made to use Decentralized Privacy-Preserving Proximity Tracing (DP-3 T) and a Temporary Contact Numbers (TCN) design, while building on the basic infrastructure developed and offered as a joint effort in the general interest by Apple and Google.^14^ A tracing design means that, on the basis of data collected by the phone, in hindsight and reasoning backwards a person’s location can be established. From a privacy viewpoint, tracing is far less intrusive than tracking. The Corona apps which are used in Europe are, therefore, tracing and not tracking apps. Initial storage of these (anonymous and pseudo-anonymous) Bluetooth data can be done centralised or decentralised, meaning that the Bluetooth codes either are stored locally on a user’s phone or immediately on a central government server. How does the app then work? Once when the application is installed on a mobile phone it needs to become active. This is done by turning on Bluetooth, enabling the app to send and receive anonymous codes from devices in its direct vicinity (1,5–2 m) which stay there for longer than 15–20 min. In case the owner of the phone is tested positive, the owner will be asked if (s)he would be willing to upload those codes to a central (government) server and, if he agrees, will be given a code which triggers the app to upload. Every active Coronavirus app will at least once, but frequently more than once, a day check with the central server whether any of the codes stored on that device were uploaded by others. If so, the app will warn its user that (s)he has been in contact with a person who has been tested positive and urges that user to have her/himself tested. Every step is voluntary, from installing the app, activating it, adding the code for upload and the decision to have yourself tested. It is completely based on the assumption that a responsible person will do this without any external constraints or pressure and it is in full accordance with the EU’s General Data Protection Regulation, as the data concerned are clearly “personal data” as referred to in the regulation. Of course, it can be asked whether in a situation of an, what at some moments seemed, out of control pandemic it might not be unavoidable to give public health concerns more priority than privacy concerns. Whenever a person’s privacy and claim to be protected in its personal and bodily integrity leads to someone else becoming ill, that other person could very well be seen as having been violated in its right to personal and bodily integrity. We then see a clash between both a fundamental citizen’s right with public interests and between two private citizens claiming the same right but in a situation where protecting one citizen’s right may harm another citizen in being protected on the basis of that very same fundamental right. Here a government, protecting public health in the interest of all, might take over and curtail individual freedom, but that choice has not been made by EU governments.

## Corona vaccination apps: identity and data certification

Using data to combat the virus, however, at a fast pace is going further than only tracing (and/or tracking) contacts of infected persons. The World Health Organisation (WHO) already introduced, many years ago, an International Certificate of Vaccination or Prophylaxis (ICVP) (“Carte Jaune” or “Yellow Card”), which contains a vaccination record. It is a medical passport that frequent travellers to countries with particular health problems will carry with them.^15^ It was particularly meant to record vaccinations against yellow fever. Given the enormous social and economic impact of the Coronavirus pandemic it can hardly come as a surprise that urgent solutions are sought making it possible in a restrained and controlled way to open up society and reinvigorate the economy. Such a solution could be that you can prove having been vaccinated against Covid-19. If evidence of vaccination could be provided, a person proving that (s)he was vaccinated could be admitted to public gatherings and be allowed to travel. The latter would be particularly relevant for international travel, but also for travel within countries where, because of the Corona pandemic, regionally differing restrictions to free movement exist. As more and more people carry a mobile phone with them it is a logical step after using that device to trace people infected with the virus that mobile phones are also used to prove that you have been inoculated. However, such an e-yellow card raises some serious issues. Who, upon inoculation, should be certified to add that information to an e-yellow card, which vaccines qualify to be registered on such a card and should a time limit be added (how long does the vaccination work)?

Until now the following has become clear regarding how, focussing on international air travel, such an immunization e-passport could work, based on information made public by the International Air Transport Association (IATA).^16^ IATA is developing a free IATA Travel Pass, which has to be downloaded to a mobile phone or tablet. The owner of the mobile phone (or tablet) then needs to take a selfie, complete a “liveness test” (moving your head, closing your eyes), scan data from the passport and the data-chip on the passport, and the app will then check whether the person holding the phone is indeed the person on the passport and that the passport is authentic. Once the information is stored on the phone a digital passport (“e-passport”) has been created. After a verified digital identity has been established further verified information on test results or on the vaccine used for vaccination could be added, following the WHO’s model for an e-yellow card. Although this all may sound rather intrusive, the app is to be built following the principle of Self-Sovereign Identity, leaving freedom to the owner of the mobile phone to share any data. However, all data that is shared voluntarily must be completely reliable and therefore verified both as to the data subject and the data object. The first phase of this process cannot but result in compulsory data sharing with, for example, governments about a citizen’s identity and national health authorities or recognised laboratories about the vaccination as such and the vaccine used. The second phase of this process, resulting in data sharing from the phone to, for example, an airline would be voluntary. However, what does in day-to-day reality voluntary mean when data sharing is a requirement for being allowed unto a flight? At the same time it should not be forgotten that international air travel already now is only possible if a traveller shows a passport or, in some cases, an identity card, to which in this new app verified information is added about vaccination. It seems only reasonable to expect that such a Corona vaccination app will facilitate international air travel.

The European Union is not preparing such an extended app as IATA, but is only focussing on an app that provides information about inoculation.^17^ Recently two regulations were published to set-up a (temporary) framework for such an app, to be called the Digital Green Certificate. One regulation concerns a framework for the issuance, verification and acceptance of interoperable certificates on vaccination, testing and recovery to facilitate free movement during the COVID-19 pandemic and the other regulation concerns a framework for the issuance, verification and acceptance of interoperable certificates on vaccination, testing and recovery to third-country nationals legally staying or legally residing in the territories of Member States during the COVID-19 Pandemic.^18^ The “Green Certificate” will give information on both data subject and data object regarding proof that a person has been vaccinated, received a negative test result or recovered from Covid-19.^19^ The certificate is meant for use within the European Economic Area (the EEA: EU and Iceland, Liechtenstein and Norway) and Switzerland. It can also be further developed as part of global initiatives; reference is made to work done by the WHO and the International Civil Aviation Organization (ICAO). Standards for travel developed by IACO are already the basis for the work by IATA in this area. Regarding the data subject a person’s name, date of birth, the issuing Member-State and a unique identifier of the certificate will be mentioned. As to the data object the certificate will contain information about the health status of the data subject, but depending upon the type of certificate. A “vaccination certificate” will contain information about the vaccine product and its manufacturer, the number of doses and the date of vaccination; a “test certificate” will provide information about the type of (negative) test, date and time of testing, the test centre and test result; a “recovery certificate” will provide information about the date of a positive test result, the issuer of the certificate that someone recovered, date of issuance and the validity date. The certificate will show an interoperable and machine readable QR (Quick Response) code that contains “necessary key data” and a digital signature of the issuing health authority (hospital, test centre) to prove the authenticity of the data.^20^ The European Commission, by building a gateway, will ensure that the certificates can be used for cross-border travel by facilitating the verification of the digital signature.

From the perspective of who actually “owns” these data, the travel app developed by IATA, the e-Yellow Card by the WHO and the Digital Green Certificate present even more complicated problems than already exist regarding data in a Covid-19 app. Both present problems about who has control over data, meaning who has access, may exclude others from access, alter, port, transfer and delete such data. With regard to vaccination passports, either as part of a travel app or as a self-standing app, a major extra problem is that the process of data collection does not begin with the phone exchanging anonymous codes with phones close by, in other words: the gathering of raw data, but starts with downloading an externally verified data object (information about, for example, vaccination) to an externally verified data subject (transforming the app into an e-passport). To put it differently: the app does not merely create data about a mobile device owner by digitally mirroring encounters in the “real” world, but it presents a copy of data already existing about the owner and which through the app are now co-generated on his or her mobile device. However, what the Covid-19 app and the Corona vaccination app have in common is that both provide very personal data about a person’s health. Who can be considered the “owner” of such data and what do we mean when we use the word “ownership” here?

## Data “ownership” and personal health data

The first question to be asked is: What is meant by data “ownership”? To begin with, the answer depends very much on the angle from which the question is being posed. In English the words “ownership” and “property” have a very wide and open meaning. The use of these words as technical terms within a legal narrative does not really change this, unless the term is legally defined in either a specific statute or case-law. A manager can be the owner of a management process, meaning that (s)he is responsible and can be held accountable. You can also be the owner of your car, meaning that it is up to your free choice to either use it or not, keep the car or sell it, but from a strictly English legal perspective ownership means that you have “title”. The latter means that you have a right to possession against any person who cannot show a better title, in other words title is relative.^21^ The statement: “This is my property!” not only refers to a physical reality (for example a house), but also to a legal reality (having the exclusive right to the house). The English terms ownership and property are, therefore, inherently ambivalent and can only be properly understood from their context.

Furthermore, it matters whether the question is being asked from the viewpoint of personal data and privacy protection (public law) or from the standpoint of marketability (private law). A public law approach will favour creating a distance from the market, not allowing parties to conclude contracts on personal data. The reason for creating such a distance is that, if a private law and, consequently, market transaction approach, would be followed, this would facilitate individual choices to contractually waive a right to privacy and data protection. Such waiver might be questionable in light of the unequal bargaining position between a private person and, for example, a tech company. More often than not between these parties an a-symmetrical access to information will exist, structurally favouring the tech company. A public law approach will be aimed at preventing the latter and in such an approach giving property entitlements (such a “ownership”) to those whose data should be protected does not fit well, because ownership would imply freedom to transfer. A private law approach, on the contrary, would begin by looking at any value exchange that might be taking place. The attitude would be that when parties voluntarily agree to give and acquire access to data, they exercise their freedom to contract, although that freedom could be limited because of data protection. Also against a private law setting great care should be taken that any information a-symmetries are being avoided or counter-balanced, however by other mechanisms than not allowing parties to conclude a contract. An example of such a counter-balance could be a mandatory right of consent withdrawal. A public law approach, therefore, would be more inclined to start by denying any “ownership” rights, whereas a private law approach would be more likely to begin by accepting that at least some type of ownership, and consequently trade in data, could exist. However, in both lines of reasoning a balance will have to be found between the public interest, aimed at protecting everyone’s personal life, and private interests, which may demand the freedom to benefit from market transactions. It is precisely this mixture of public and private interests which is giving rise to growing acceptance of the idea that rights regarding data (“data rights”) might very well be both: In some respects public and in some other respects private.^22^

Still, we should not miss an essential point here: the nature of data as such. From a legal perspective data are non-defined, non-categorised and non-specified objects. To begin with, already serious difficulties arise regarding how to define data as such and whether this should be a technical, legal or mixed technical-legal definition. Defining data is like defining air, water and sunshine: the latter are part of our physical environment and we know how to delineate them, but from a legal viewpoint that is not sufficient to make air, water and sun an object which private law can target. Accepting that next to our physically reality we are now also surrounded by a virtual environment, does not make data a targetable legal object. In order for the law being able to regulate, data must be categorised and, for purposes of private law, even specified. Categorisation can be done on the basis of whether data are connected with a person (personal data) or not (non-personal data), but also on the basis of how data are gathered and processed (for example raw, derived, resulting, inferred and big data).^23^ For market transactions even this is not sufficient, more specification will then be needed, a major problem being the non-rivalrous and non-depletable nature of data: they can be limitlessly copied. Particularly to counter their non-rivalrous nature specification can be done in two ways: physical and non-physical. Physical by delineating the data object on the basis of their physical container (USB stick, server) or by using software that creates limits to copying.^24^ The latter can be done through Distributed Ledger Technology (blockchain) creating unchangeable data “blocks” and making a copy of these blocks pointless because of the control that the technology has over individual blocks through ledger maintained and computer (“nodes”) governed peer-to-peer transfers in the blockchain’s ecosystem. But even when data can be specified the fact that, though perhaps limited, copies may exist creates its own problems. Traditionally, the Civil Law only accepts ownership when, ideally, there is one subject having the most extensive, exclusive right, unlimited in time, over an object against all other subjects. This paradigm cannot be applied to data. In other words: the above, briefly sketched debate, about whether to approach data ownership from the perspective of public law or private law, is pointless. We need to rethink, in light of the very nature of data, what we mean by “ownership”. The conclusion can then only be that data ownership is fundamentally different from ownership of physical assets such as a car, or a house. At the end of the day we will have to accept that it is management, nothing more, nothing less.

## Data “ownership” as management

A fundamental comparative legal analysis as to who might have a property entitlement to data will have to focus on four aspects. These are: (1) who would be the subject of such entitlement, (2) who would belong to the considerable and relevant group of other subjects against whom the right can be effected, (3) what would be the object of such a right and, of course, (4) the content of the right itself. Traditionally, the Civil Law distinguishes between “personal” and “real” rights, but we already saw that in more recent scholarship a third category is introduced called “data rights”, which, depending upon the angle from which these rights are being observed, traditionally would be called quasi-personal or quasi-real. What we gradually begin to realise is that dogmatic distinctions which helped us create a systematic and coherent system of property law, facilitating both legislatures and courts to solve societal problems in a rationalised and non-arbitrary way, are deeply rooted in a what might be called “analogue” world. In today’s hybrid society these old categories still satisfactorily serve the very important purpose of being a point of reference for creating a new stable and predictable legal governance structure that proves to be effective. Dogmatic legal thinking carries with it acquired wisdom, which should not be too easily discarded. At the same time, such thinking now meets its limits when applied to our developing hybrid world. We need to revisit our existing legal categories, test these categories to find out if, perhaps after some adaptation, they still might function adequately, or should be left aside and new categories should be developed. Considering the above, adding to the fundamental distinction of subjective rights the category of data rights is a very helpful step forward. The question, however, remains if what we are doing goes far enough. In a certain way history is repeating itself. When we did not know how to deal with the legal aspects of legal creativity, a whole new area of property law was created called “intellectual property law”. By thus creating, as it were, a new legal object, we could then apply the other elements of the legal-analytical framework of property law without too many problems. The same subjects as in traditional, general, property law could be subject in the sense of right holder, a right that looking at its content closely resembled existing property rights and could be applied against such a considerable and relevant group of other subjects that all aspects of a “real” right were again fulfilled. Are not we now doing the same with data? Not quite. Regarding data we are faced not only with a very different nature of the object, but also with questions regarding who could be subject/right holder, against whom could such a right be invoked and to what degree is the content of the right not only strictly private, but also public? Regarding subjects as right holders, it can be debated whether an autonomously functioning, Artificially Intelligent driven, computer system might not be seen as a legal person in the same sense as, for example, a corporation. Traditional property law assumes that the group of persons against whom a right can be invoked is stable in the sense that this group can be pre-defined in a static way. A right of mortgage can be invoked against the owner of a house and against other holders of property rights which do not precede that right (prior tempore rule). But could not it be that, given the fluid, non-rivalrous and non-depletable nature of data, we are now confronted with a continuously floating object demanding that we also face a floating group of third parties? Furthermore, because data are both a mirror of our social reality (and of us as its citizens) and a creator of that same reality, the old distinction between private law and public law no longer holds. From a comparative, and legal-historical, aspect these questions cannot really be surprising. The Common Law tradition never had this strict separation between personal and real rights: these were more notions than hard and fast categories. In this tradition a separation between private and public law only came later than in the Civil Law. From an historical-ideological viewpoint it is interesting to note that in the Marxist view of the law, the role of the state and the role of the individual area also not separate.^25^ And even for the Civil Law tradition the questions are not really as disturbing as they might seem. Already in 1834 the French Supreme Court in its Caquelard c. Lemoine ruling decided that the definition of ownership in article 544 of the French Civil Code had not abolished other pre-existing types of ownership, such as the customary property law of Normandy.^26^ Some years ago that decision was re-affirmed in two cases regarding the Maison de Poésie, about whether a new property right (a property right of special use) could be accepted.^27^ It is also interesting to note that in the recently enacted reform of Belgian property law also a less dogmatic approach to property law and more particularly the numerus clausus can be found.^28^

Following the analysis advocated above the following will offer an overview of the various elements of property rights, applied to Covid-19 apps and Corona vaccination apps, in order to provide a more concrete examination.

- A multi-perspective view
  Data rights, as described earlier, are a multi-perspective type of rights, in a borderline region in between private and public law and in the middle of personal and real rights. The latter type of rights can, if data rights were to be accepted by legislatures and courts, be considered as representing single-perspective rights. Looking at their content, data rights provide control, both positive and negative, rather similar to traditional property rights. Positive control, as they provide their holder with the power of access, use, modification, erasure, portability and transferability. Negative control, because they allow others to be excluded and prevented from exercising these positive powers. What, furthermore, should be added—and this might prove to be a very serious problem of practical application—is that this multi-perspectivity is not static, but dynamic and in its final outcome resembles more a (de facto) management than a (de iure) entitlement approach.^29^ In any case, a data rights approach at least mitigates a “might is right” attitude. The data rights of a person will change depending upon other persons also gaining some form of control, which more often than not will be the case as several copies of data may exist to which a number of people at the same time may have varying degrees of access. That control can be of a personal nature, but also be used for commercial purposes or in the general interest. On the whole, data rights resemble more a legal status than a primary property right such as “ownership” in the Civil Law tradition. By legal status in this context I mean a relative position at a particular point in time.^30^ What the right contains depends upon the rights of others who can manage the data and this may (and most likely will) fluctuate as time moves on and positions of managers change. Let me explain this further. As to the status of the manager we can distinguish primary managers (“data owners”), such as the data producer, in the case of tracing apps the citizen and in the case of the vaccination app a government with regard to a person’s identity and a public health institution concerning for example the vaccination. Next to primary managers there are secondary managers (“data stewards”), for instance the government in the interest of public health as can be seen following the upload of Bluetooth codes in Covid-19 apps to a government server after someone was tested positive. Finally, we can distinguish tertiary managers (“data users”), for example in the case of tracing apps one can think of health care providers, academic researchers, the pharmaceutical industry and in case of vaccination apps for example the person who wants to use the app for travel, the airline industry or organisers of mass meetings. It is interesting to note that more and more governments seem to consider themselves being entitled to data management as an expression of their sovereign rights. In the future this might result in a claim to ultimate entitlement (comparable to the doctrine of “dominium eminens”), justifying that a government expropriates data by obligatory sharing of data for example in the interest of public health.
- Subject who is a right-holder
  From the viewpoint that entitlement (“ownership”) of data is more a matter of management than entitlement, we can differentiate between various stakeholders who can be given one of the above mentioned types of management rights. In the case of Covid-19 apps stakeholders are Individual citizens, governments (health authorities), medical care providers (not only family doctors and hospitals, but also health insurers), scientific researchers and the pharmaceutical industry. Regarding Corona vaccination apps the stakeholders are even more diverse. Next to, again, individual citizens, governments and medical health providers, also passenger transport services (railway and bus enterprises, airlines), organisers of public events, and the tourism and hospitality industry (hotels, restaurants) are interested parties. The difficulty is that the interests of all these parties do not come into existence at the same time and their interest, which will be foremost access to data, is effected by providing them with a copy of the data. The more copies of data exist the less it becomes possible to control data. This is why primary managers are those subjects who “produce” or “create” the data. In the examples which I am using these are individual citizens who have the Covid-19 app installed on their mobile phone and health authorities responsible for the vaccination. Both with regard to the individuals and to the health authorities it can be said that they create the data. Citizens by coming into close contact with others for a longer period and health authorities by vaccinating people. The very moment the data are shared the situation already changes. In the case of Covid-19 apps if you are tested positive, you will be asked to upload your data to a central government computer. At that moment the individual loses full control. The same applies when the data in a Corona vaccination app are shared by the individual who has been inoculated. Data sharing means that data control is now also shared. In other words: the position of the data subjects changes over time, depending upon who else now has a copy of the data object.
- Subjects who can be excluded
  As long as data are not shared by copying them, any other subject can be excluded from control over these data. It does, however, matter how subjects who are first excluded later on gain control over data, be it by receiving a copy or by merely getting access. If control is given to others on a voluntary basis, the subject who now has a copy will from that moment on also have certain (perhaps all) rights concerning the data which the original data subject had. But especially in a situation of personal data in the sense of the GDPR the original data subject could very well retain a degree of initial control. The processing of personal data is only allowed in as far as this remains within the purpose for which the data subject gave its consent.^31^ However, such a consent can, under the regime of the GDPR, be withdrawn.^32^ The situation is, of course, completely different in case data is stolen. A hacker has control over data, but does not have a right that flows from such control. Being in control, however, undeniably gives an illegitimate manager a bargaining position, but no legally enforceable rights will come into existence, unless perhaps when a third party in good faith gets access to such data. However, these are questions which demand a separate analysis.
- Object
  Regarding the object it has already been remarked that data are essentially different from the traditional categories of legal objects. Historically, private law focussed on physical assets and later on intangibles, particularly monetary claims. Intellectual property became a legal object with its own field. We now see the same happening with regard to data.
- Content of the right
  The content of the right has, as is the case with traditional property rights, a positive and a negative aspect. Looking at it from a positive side, the position of the subject/right-holder depends upon the rights of other subjects/right-holders. From a negative side it means that the possibility to exclude others is also relative. Any stakeholder who has control over data will in the legal exercise of that control be dependent on its standing vis-à-vis other stakeholders with control. With any new stakeholder the position of all others changes. The content of the particular data right is therefore not static, but dynamic. Also the nature of the data will be relevant and also this nature can change. Under EU law personal data are treated in a fundamentally different way from non-personal data. If any data are personal the data subject is entitled to far-reaching data protection. However, by giving consent this data protection can be waived. Concerning non-personal data the EU has a policy of promoting the free flow of such data, creating a fifth fundamental market freedom next to the freedoms of persons, goods, services and capital.^33^ Consequently, depending on the nature of the data and whether consent is given to process personal data again the content of the data right of stakeholders changes. Data rights are, therefore, not so much entitlements, but they resemble more a position of management; they are not only multi-perspective, they are profoundly dynamic.

## Concluding remarks

Already before the present Covid-19 health crisis an emerging trend could be seen towards offering health services from a distance, called “e-health”. This trend, like so many other developments towards digitalisation of our societies, received a considerable impetus because of the Covid-19 crisis. First, the rise of Covid-19 tracing (and/or tracking) apps and now to be followed by the advance of Corona vaccination apps has made us aware of the benefits which e-health may bring, particularly in a situation where distance means safety. The apps contain very personal information and, consequently, have provoked questions as to whether the apps sufficiently protect a person’s right to privacy and data protection as safeguarded by the EU’s General Data Protection Regulation. The nature of the data, however, is such that also questions as to the importance of access by public health authorities in the public interest can be asked. Also, although commercial, but still important for developing and producing vaccines, for the pharmaceutical industry the data are important. The result is a conflict particularly between entitlement to privacy protection and the general interest, causing questions to be asked about which interest has priority. It might very well be, however, that this question, asked as such, is beside the point. Given that data are non-rivalrous and non-depletable, because they can be copied and copied, questions about which entitlement has priority cannot be answered in absolute terms. Rights regarding data depend upon who at a particular time has control over the data, who else has control and what control between all those involved then means. Looking at who has which right to data one can see an entitlement paradigm surfacing which is multi-perspective, relative and dynamic. Calling data entitlement “ownership” is not a reference to ownership in the traditional sense of the word, but to management. To decide what management in a particular situation means interest balancing exercises must be made. These exercises will change over time, as accordingly will the answer to the question who is “owner” of data in Covid-19 and Corona vaccination apps.

## Footnotes

1 Cf. for these developments from an EU governance aspect: European Commission Staff Working Document on Better regulations for innovation-driven investment at EU level (Luxemburg: European Union, 2016), p. 17 ff., Regulation (EU) 2017/745 of the European Parliament and of the Council of 5 April 2017 on medical devices, amending Directive 2001/83/EC, Regulation (EC) No 178/2002 and Regulation (EC) No 1223/2009 and repealing Council Directives 90/385/EEC and 93/42/EEC, OJ 2017, L 117/1 and the Proposal for a Regulation of the European Parliament and of the Council on health technology assessment and amending Directive 2011/24/EU, Brussels, 22 December 2020, Interinstitutional File: 2018/0018(COD). It is interesting to note here recital 19 of Regulation (EU) 2017/45, stating on whether software can qualify as a medical device: “It is necessary to clarify that software in its own right, when specifically intended by the manufacturer to be used for one or more of the medical purposes set out in the definition of a medical device, qualifies as a medical device, while software for general purposes, even when used in a healthcare setting, or software intended for life-style and well-being purposes is not a medical device. The qualification of software, either as a device or an accessory, is independent of the software's location or the type of interconnection between the software and a device.”

2 Regarding Covid-19 apps see. Sjef van Erp ( 2020a, b), ‘Who 'Owns' the Data in a Coronavirus Tracing (and/or Tracking) App?’ in: Ewoud Hondius, Marta Santos Silva, Andrea Nicolussi, Pablo Salvador Coderch, Christiane Wendehorst & Fryderyk Zoll (eds.), *Coronavirus and the Law in Europe*, also available online at: https://www.intersentiaonline.com/bundle/coronavirus-and-the-law-in-europe and https://papers.ssrn.com/sol3/papers.cfm?abstract_id=3678095 (with further references).

3 The debate on whether data can be “owned” has given rise to considerable debate. See, as an example, a recent blogpost by Dirk Auer (2021), The Problem of Data Property Rights, discussing personalized medicine and medical data, to be found at: https://truthonthemarket.com/2021/03/23/the-problem-of-data-property-rights/.

4 Lian Yuming (ed.) (2019), Data Rights Law 1.0, The Theoretical Basis. Key Laboratory of Big Data Strategy (Oxford: Peter Lang, 2019), examining in Chapter 3 the concept of data rights.

5 More information about the ALI-ELI project on Principles for a Data Economy can be found on the project website: https://principlesforadataeconomy.org/.

6 John Thomas and Christiane Wendehorst (2020), Response to the public consultation on “A European strategy for data” COM(2020) 66 final, p. 6, to be found on the website of the European Law Institute: https://www.europeanlawinstitute.eu/fileadmin/user_upload/p_eli/Projects/Data_Economy/ELI_Response_European_Strategy_for_Data.pdf and the blogposts by Charlotte Ducuing (2020a, b), ‘Data rights in co-generated data’: The ground-breaking proposal under development at ELI and ALI, https://www.law.kuleuven.be/citip/blog/data-rights-in-co-generated-data-part-1/ and ‘Data rights in co-generated data’: How to legally qualify such a legal ‘UFO’?, https://www.law.kuleuven.be/citip/blog/data-rights-in-co-generated-data-part-2/. The Response by Thomas and Wendehorst continues: “Data is a non-rivalrous resource, which may be used by many different parties for many different purposes at the same time, and to the generation of which many parties may have contributed in many different ways These attributes are taken into account when recommending the recognition of a new data-specific class of rights, which the ALI-ELI Principles call ‘data rights.’ Rights of this nature are being recognized to an increasing extent in data-specific legislation and case law worldwide. They are not purely contractual, as they may exist between parties without any contractual link, and they do not reflect ownership notions in the traditional sense because traditional notions of ownership do not work well with resources of a non-rivalrous nature. The data rights dealt with under the ALI-ELI Principles fulfil functions similar to those fulfilled by ownership with regard to traditional rivalrous assets. However, the notion of data rights recommended by the ALI-ELI Principles is not identical to that of ownership rights. While the right to control a resource as against any person who has a lesser right is central to ownership in the classical sense, these Principles take the position that the right to have non-exclusive access to data or to port data is central to any equivalent of the concept of ownership in the data economy, not least because the overall welfare is normally increased where more than one person can exploit the data for economic purposes. The ALI-ELI Principles set out a non-exclusive list of four basis data rights: (1) Access or Porting of co-generated data. (2) Desistance from the use of co-generated data. (3) Correction of co-generated data. (4) Economic share in profits derived from co-generated data.”

7 Merriam-Webster dictionary: https://www.merriam-webster.com/dictionary/object.

8 Cf. Sjef van Erp (2017), Ownership of Data: The Numerus Clausus of Legal Objects, Brigham-Kanner Property Rights Conference Journal 2017, p. 235–257.

9 The full text of the Act can be found at: https://www.legislation.gov.au/Details/C2020A00044.

10 Communication from the Commission to the European Parliament, the Council, The European Economic and Social Committee and the Committee of the Regions. 2030 Digital Compass: the European way for the Digital Decade, Brussels, 9.3.2021, COM(2021) 118 final.

11 See also the report by EIT Digital, European Digital Infrastructure and Data Sovereignty—A Policy Perspective (Brussels: EIT Digital, 2020). In this report briefly the differences in approach between the EU, USA and China are discussed.

12 Cf. the Communication Towards a common European data space SWD(2018) 125 final, COM(2018) 232 final) and the Communication A European strategy for data (COM(2020) 66 final).

13 Regulation (EU) 2016/679 of the European Parliament and of the Council of 27 April 2016 on the protection of natural persons with regard to the processing of personal data and on the free movement of such data, and repealing Directive 95/46/EC (General Data Protection Regulation), OJ 2016, L 119/1. Article 4(1) GDPR reads as follows: “‘personal data’ means any information relating to an identified or identifiable natural person (‘data subject’); an identifiable natural person is one who can be identified, directly or indirectly, in particular by reference to an identifier such as a name, an identification number, location data, an online identifier or to one or more factors specific to the physical, physiological, genetic, mental, economic, cultural or social identity of that natural person;”.

14 More technical information can be found in a European Parliament briefing paper: National COVID-19 contact tracing apps, to be found at https://www.europarl.europa.eu/RegData/etudes/BRIE/2020/652711/IPOL_BRI(2020)652711_EN.pdf.

15 See https://www.who.int/ihr/ports_airports/icvp/en/.

16 Cf. the information made available by the International Air Transport Association (IATA): https://www.iata.org/en/pressroom/pr/2020-12-16-01/.

17 It should, however, not be forgotten that within the European Union already a scheme exists that enables mutual recognition of electronic identification: the eIDAS regulation: Regulation (EU) No 910/2014 of the European Parliament and of the Council of 23 July 2014 on electronic identification and trust services for electronic transactions in the internal market and repealing Directive 1999/93/EC, OJ 2014, L 257/73. Furthermore, the European Commission announced its intention to create a secure e-Identity for all European citizens, see the earlier mentioned 2030 Digital Compass.

18 Proposal for a Regulation of the European Parliament and of the Council on a framework for the issuance, verification and acceptance of interoperable certificates on vaccination, testing and recovery to facilitate free movement during the COVID-19 pandemic (Digital Green Certificate), Brussels, 17.3.2021, COM(2021) 130 final, 2021/0068 (COD) and Proposal for a Regulation of the European Parliament and of the Council on a framework for the issuance, verification and acceptance of interoperable certificates on vaccination, testing and recovery to third-country nationals legally staying or legally residing in the territories of Member States during the COVID-19 pandemic (Digital Green Certificate), Brussels, 17.3.2021, COM(2021) 140 final, 2021/0071 (COD). For a European Parliament briefing paper on the Digital Green Certificate see https://www.europarl.europa.eu/RegData/etudes/ATAG/2021/690531/EPRS_ATA(2021)690531_EN.pdf. The paper stresses the support given to the initiative by stakeholders from the tourism and transport sectors.

19 See https://ec.europa.eu/commission/presscorner/detail/en/qanda_21_1187.

20 See the European Commission’s website on the Green Digital Certificate: https://ec.europa.eu/commission/presscorner/detail/en/qanda_21_1187.

21 Sjef van Erp and Bram Akkermans (eds.) (2012), Cases, Materials and Text on Property Law, Ius Commune Casebooks for the Common Law of Europe (Oxford and Portland, Or.: Hart Publishing, 2012), p. 202–203.

22 Cf. the Principles for a Data Economy, a joint project of the American Law Institute (ALI) and the European Institute (ELI), which takes “data rights” as an overarching concept to explain and create legal interests in data.

23 See Sjef van Erp (2017), Ownership of data and the numerus clausus of legal objects, in: United Nations Commission on International Trade Law (UNCITRAL), Modernizing International Trade Law to Support Innovation and Sustainable Development. Proceedings of the Congress of the United Nations Commission on International Trade Law, Vienna, 4–6 July 2017,Volume 4: Papers presented at the Congress (Vienna: United Nations, 2017), p. 69–80.

24 This same idea of the need to have a “data container” before any further legal-analytical steps can be taken, is also touched upon, but from a more general data governance viewpoint, in a recent report by EIT Digital, European Digital Infrastructure and Data Sovereignty. A policy perspective (Brussels: EIT Digital, 2020), p. 20 and 21.

25 Cf. Jean Axelrad Cahan (1995), The Concept of Property in Marx's Theory of History: A Defense of the Autonomy of the`Socioeconomic Base, Science & Society, 1994–1995, p. 392–414.

26 Caquelard c. Lemoine, Req. 13 februari 1834, D. 1834, 1, 218, S. 34, 1, 205.

27 Maison de Poésie I, Cass Civ 3ème 31 October 2012, no. 11–16,304 and Maison de Poésie II, Cass. 3ème civ. 8 September 2016, n° 14–26.953.

28 The new property law provisions can be found in the new Book 3 Belgian Civil Code. The text, which will enter into force on 1 September 2021, is available electronically: http://www.ejustice.just.fgov.be/cgi_loi/change_lg.pl?language=nl&la=N&cn=2020020416&table_name=wet (Dutch version), http://www.ejustice.just.fgov.be/cgi_loi/change_lg.pl?language=fr&la=F&cn=2020020416&table_name=loi (French version).

29 See Sjef van Erp (2020a, b), Management as Ownership of Data, in: Sebastian Lohsse, Reiner Schulze and Dirk Staudenmayer (eds.), Data as Counter-Performance—Contract Law 2.0? (Baden-Baden: Nomos, 2020), p. 77–93.

30 When referring to status against a legal setting, often Henry Maine’s famous phrase is quoted that “the movement of the progressive societies has hitherto been a movement from Status to Contract”. See Henry Sumner Maine (1906), Ancient Law, Its connection with the early history of society and its relations to modern ideas (with Introduction and Notes by Sir Frederick Pollock; London: John Murray, 1906), p. 174, critically analysed by Katharina Isabel Schmidt (2017), Henry Maine’s “Modern Law”: From Status to Contract and Back Again?, The American Journal of Comparative Law 2017, p. 145–186.

31 Article 6(4) GDPR.

32 See articles 6(1)(a) and 7(3) GDPR.

33 See Regulation (EU) 2018/1807 of the European Parliament and of the Council of 14 November 2018 on a framework for the free flow of non-personal data in the European Union, [2018] OJ L303/59.
